# Novel subtypes of severe COVID-19 respiratory failure based on biological heterogeneity: a secondary analysis of a randomized controlled trial

**DOI:** 10.1186/s13054-024-04819-0

**Published:** 2024-02-21

**Authors:** Narges Alipanah-Lechner, James Hurst-Hopf, Kevin Delucchi, Lamorna Swigart, Andrew Willmore, Benjamin LaCombe, Robin Dewar, H. Clifford Lane, Perrine Lallemand, Kathleen D. Liu, Laura Esserman, Michael A. Matthay, Carolyn S. Calfee, the I-SPY COVID Consortium, Neil R. Aggarwal, Timothy Albertson, Sara Auld, Jeremy R. Beitler, Paul Berger, Ellen L. Burnham, Nathan Cobb, Alessio Crippa, Andrea Discacciati, Martin Eklund, D. Clark Files, Eliot Friedman, Sheetal Gandotra, Kashif Khan, Jonathan Koff, Santhi Kumar, Thomas R. Martin, Nuala J. Meyer, Timothy Obermiller, Philip Robinson, Derek Russell, Karl Thomas, Se Fum Wong, Richard G. Wunderink, Mark M. Wurfel, Albert Yen, Fady A. Youssef, Anita Darmanian, Amy L. Dzierba, Ivan Garcia, Katarzyna Gosek, Purnema Madahar, Aaron M. Mittel, Justin Muir, Amanda Rosen, John Schicchi, Alexis L. Serra, Romina Wahab, Kevin W. Gibbs, Leigha Landreth, Mary LaRose, Lisa Parks, Adina Wynn, Caroline A. G. Ittner, Nilam S. Mangalmurti, John P. Reilly, Donna Harris, Abhishek Methukupally, Siddharth Patel, Lindsie Boerger, John Kazianis, Carrie Higgins, Jeff McKeehan, Brian Daniel, Scott Fields, Alejandra Jauregui, Daniel Belvins, Catherine Nguyen, Alexis Suarez, Maged A. Tanios, Farjad Sarafian, Usman Shah, Max Adelman, Christina Creel-Bulos, Joshua Detelich, Gavin Harris, Katherine Nugent, Christina Spainhour, Philip Yang, Angela Haczku, Erin Hardy, Richart Harper, Brian Morrissey, Christian Sandrock, G. R. Scott Budinger, Helen K. Donnelly, Benjamin D. Singer, Ari Moskowitz, Melissa Coleman, Joseph Levitt, Ruixiao Lu, Paul Henderson, Adam Asare, Imogene Dunn, Alejandro Botello Barragan

**Affiliations:** 1 Division of Pulmonary, Critical Care, Allergy, and Sleep Medicine, University of California, Room M-1083, 505 Parnassus Ave., San Francisco, CA 94143 USA; 2 Department of Psychiatry and Behavioral Sciences, University of California, San Francisco, CA USA; 3 Department of Laboratory Medicine, University of California, San Francisco, CA USA; 4 Cardiovascular Research Institute, University of California, San Francisco, CA USA; 5 Virus Isolation and Serology Laboratory, Applied and Developmental Directorate, Frederick National Laboratory, Frederick, MD USA; 6 Division of Clinical Research, National Institute of Allergy and Infectious Diseases, National Institutes of Health, Bethesda, MD USA; 7 Division of Nephrology, University of California, San Francisco, CA USA; 8 Department of Anesthesia, University of California, San Francisco, CA USA; 9 Department of Surgery, University of California, San Francisco, CA USA

**Keywords:** COVID-19, Hypoxemic respiratory failure, Latent class analysis, Phenotyping, Biological heterogeneity, Protein biomarkers

## Abstract

**Background:**

Despite evidence associating inflammatory biomarkers with worse outcomes in hospitalized adults with COVID-19, trials of immunomodulatory therapies have met with mixed results, likely due in part to biological heterogeneity of participants. Latent class analysis (LCA) of clinical and protein biomarker data has identified two subtypes of non-COVID acute respiratory distress syndrome (ARDS) with different clinical outcomes and treatment responses. We studied biological heterogeneity and clinical outcomes in a multi-institutional platform randomized controlled trial of adults with severe COVID-19 hypoxemic respiratory failure (I-SPY COVID).

**Methods:**

Clinical and plasma protein biomarker data were analyzed from 400 trial participants enrolled from September 2020 until October 2021 with severe COVID-19 requiring ≥ 6 L/min supplemental oxygen. Seventeen hypothesis-directed protein biomarkers were measured at enrollment using multiplex Luminex panels or single analyte enzyme linked immunoassay methods (ELISA). Biomarkers and clinical variables were used to test for latent subtypes and longitudinal biomarker changes by subtype were explored. A validated parsimonious model using interleukin-8, bicarbonate, and protein C was used for comparison with non-COVID hyper- and hypo-inflammatory ARDS subtypes.

**Results:**

Average participant age was 60 ± 14 years; 67% were male, and 28-day mortality was 25%. At trial enrollment, 85% of participants required high flow oxygen or non-invasive ventilation, and 97% were receiving dexamethasone. Several biomarkers of inflammation (IL-6, IL-8, IL-10, sTNFR-1, TREM-1), epithelial injury (sRAGE), and endothelial injury (Ang-1, thrombomodulin) were associated with 28- and 60-day mortality. Two latent subtypes were identified. Subtype 2 (27% of participants) was characterized by persistent derangements in biomarkers of inflammation, endothelial and epithelial injury, and disordered coagulation and had twice the mortality rate compared with Subtype 1. Only one person was classified as hyper-inflammatory using the previously validated non-COVID ARDS model.

**Conclusions:**

We discovered evidence of two novel biological subtypes of severe COVID-19 with significantly different clinical outcomes. These subtypes differed from previously established hyper- and hypo-inflammatory non-COVID subtypes of ARDS. Biological heterogeneity may explain inconsistent findings from trials of hospitalized patients with COVID-19 and guide treatment approaches.

**Supplementary Information:**

The online version contains supplementary material available at 10.1186/s13054-024-04819-0.

## Background

Coronavirus disease 2019 (COVID-19) is clinically heterogeneous, ranging from asymptomatic disease to protracted critical illness with a high fatality rate [1]. Variability in the severe acute respiratory syndrome coronavirus-2 (SARS-CoV-2), the environment, and the host all contribute to this heterogeneity. Host-related clinical factors associated with poor outcomes include male sex, obesity, older age, and the presence of comorbidities such as diabetes mellitus and hypertension [2, 3]. Studies on the host immunologic response to SARS-CoV-2 have found associations between circulating levels of interleukin- (IL-) 6, tumor necrosis factor (TNF), interferon- (IFN-) α, IL-10 and other cytokines with outcomes in those with moderate to severe disease [4]. Trials of immunomodulators in this population, however, have met with mixed results, likely in part due to complex host–pathogen interactions. For instance, the RECOVERY trial found that dexamethasone led to a reduction in 28-day mortality in severe COVID-19 [5]. However, four other trials reached different conclusions [6–9]. Similar findings have been reported with IL-6 inhibitors [10–15] and the antiviral therapy, remdesivir [16–19]. These disparate findings suggest that trial participants may be biologically heterogeneous, leading to studies that are underpowered to detect meaningful treatment effects.

Latent class analysis (LCA) of clinical and protein biomarker data has identified two subtypes of non-COVID acute respiratory distress syndrome (ARDS), termed “hyper-inflammatory” and “hypo-inflammatory,” the former characterized by elevated plasma levels of inflammatory biomarkers such as IL-6 and soluble TNF receptor 1 (sTNFR-1) and lower levels of bicarbonate and protein C [20]. These subtypes have significantly different mortality in secondary analyses of numerous trials and observational studies [20–24] Moreover, the subtypes demonstrate different responses to therapies such as PEEP, fluid strategy, and simvastatin in secondary analyses of randomized trials [20, 21, 24]. A similar approach to subtyping patients with COVID-19 ARDS early in the pandemic revealed significant overlap with non-COVID ARDS subtypes, but with the exception of IL-6, data on biomarkers of lung injury and inflammation were unavailable [25]. Additionally, corticosteroids were associated with reduced 90-day mortality in one subtype, offering a plausible explanation for the mixed results from corticosteroid trials in severe COVID-19 [26]. An observational study similarly found two distinct subtypes of COVID-19 ARDS using clinical data from a single center, early in the pandemic. No data on biomarkers of lung injury or inflammation were collected [27]. A recent retrospective cohort study by Verhoef et al. used latent profile analysis of clinical and extensive protein biomarker data from a cohort of patients with COVID-19 who presented to acute care facilities and identified two novel subtypes with heterogeneous treatment response to corticosteroids [28]. Due to the observational nature of the recruited cohort, the study was limited by heterogeneous practices around steroid or other immunomodulator drug administration, timing of SARS-CoV-2 infection, and a mix of patients from the outpatient and inpatient settings, limiting the conclusions that can be drawn with regards to the biology of patients with severe COVID-19.

In this study, the primary objective was to apply LCA using clinical and protein biomarker data to test for evidence of latent subtypes in the I-SPY COVID randomized controlled trial of investigational agents for the treatment of severe COVID-19. [29]

## Methods

### Study design

Data were obtained from the I-SPY COVID Trial, a multi-institutional phase 2 platform randomized controlled open-label trial to evaluate pharmacotherapies for severe COVID-19 (NCT04488081). The trial protocol and results for the first seven agents, none of which met predefined criteria for benefit, have been reported [29, 30]. In brief, newly hospitalized adults with SARS-CoV-2 requiring ≥ 6 L/min supplemental oxygen were randomized to either a control arm receiving backbone therapy (Remdesivir and dexamethasone) or an investigational arm receiving backbone therapy plus a study drug.

### Assay procedures

Plasma was collected on days 1 (baseline, prior to administration of any active study drug), 3, and 7 following trial enrollment. Seventeen candidate protein biomarkers of inflammation, disordered coagulation, endothelial, and epithelial lung injury were selected a priori based on evidence from non-COVID ARDS and early evidence from COVID-19 studies. Biomarkers were measured using customized and validated multiplex Luminex panels or in duplicate using single analyte enzyme linked immunoassay methods (ELISA) (Additional file 1: methods). SARS-CoV-2 nucleocapsid antigen levels were measured using a single-molecule immune bead assay (Quanterix, Billerica, MA, USA).

### Statistical analysis

The primary outcome for these analyses was 28-day mortality. Secondary outcomes included 60-day mortality, time to death, and time to recovery. The association of each biomarker with mortality was tested using unadjusted logistic regression models and models adjusting for age, BMI, and level of respiratory support required at study enrollment. P-values were adjusted for multiple comparisons using False Discovery Rate (FDR) cutoff of 0.05. The Fine-Gray model for cumulative incidence function was used to estimate a subdistribution hazard ratio (SHR) for the association of each biomarker with time to death and time to recovery, as recovery and death were competing events in the trial (Additional file 1: methods).

A set of clinical and protein biomarker data were selected a priori to serve as subtype defining variables for LCA. Modeling was performed agnostic of clinical outcomes using standard methods [31]. A validated parsimonious model using IL-8, bicarbonate, and protein C was used for comparison with established hyper- and hypo-inflammatory LCA subtypes of non-COVID ARDS [32]. Using longitudinal biomarker measurements in the control arm, we fit linear mixed effects models to determine whether subtype assignment was associated with differences in biomarker trajectory over time.

Analyses were conducted in R 4.2.2, STATA 17.0, and MPlus 8.8.

## Results

### Cohort characteristics

Of 868 patients randomized in the I-SPY COVID trial from September 18th, 2020, until October 6th, 2021, 597 had plasma collected at baseline (Additional file 2: Figure S1). The first 400 were included in the analyses to provide adequate power for LCA analyses [33, 34]. Average age was 60 ± 14 years; 67% were male, and 28-day mortality was 25% (Table 1). At trial enrollment, 26% required positive pressure ventilation, and 97% were receiving dexamethasone. Patients excluded from these analyses had similar characteristics to those included (Additional file 2: Table S1).

**Table 1:** Characteristics of the study population and outcomes by assigned I-SPY COVID trial arm

	Control arm (N = 142)	Investigational arm (N = 258)	*P* value*
Age (mean ± SD)	59.3 ± 14.4	60.1 ± 14.0	0.6
Male sex	93 (66%)	174 (67%)	0.78
BMI (median (IQR))	32 (28 to 39)	32 (28 to 36)	0.26
*Race*			0.38
Black or African American	38 (27%)	51 (20%)	
White or Caucasian	73 (51%)	141 (55%)	
Other	8 (6%)	21 (8%)	
Unknown	23 (16%)	45 (17%)	
*Ethnicity*			0.17
Hispanic/Latinx	42 (30%)	69 (27%)	
Not Hispanic/Latinx	98 (69%)	176 (68%)	
Unknown	2 (1%)	13 (5%)	
*Comorbidities*			
Cerebrovascular disease	8 (6%)	13 (5%)	0.98
Congestive heart failure	11 (8%)	14 (5%)	0.48
Diabetes	56 (39%)	87 (34%)	0.3
Chronic kidney disease	21 (15%)	25 (10%)	0.22
End stage kidney disease	1 (0.7%)	1 (0.4%)	1
Dialysis	0 (0%)	2 (0.8%)	0.76
Hypertension	84 (59%)	144 (56%)	0.59
Liver disease—mild	2 (1%)	3 (1%)	1
Liver disease—moderate to severe	1 (0.7%)	1 (0.4%)	1
Myocardial infarction	5 (3%)	6 (2%)	0.7
Peripheral vascular disease	7 (5%)	6 (2%)	0.27
Chronic Lung disease	30 (21%)	49 (19%)	0.7
Chronic rheumatologic disease	8 (6%)	13 (5%)	0.98
*COVID-19 severity—enrollment*^*†*^			0.58
WHO 5	117 (82%)	221 (86%)	
WHO 6	12 (9%)	15 (6%)	
WHO 7	13 (9%)	22 (9%)	
*Respiratory support, enrollment*			0.84
≤ 15 LPM oxygen	14 (10%)	28 (11%)	
> 15 LPM oxygen	90 (63%)	165 (64%)	
Noninvasive mechanical ventilation	13 (9%)	27 (11%)	
Invasive Mechanical ventilation	25 (18%)	37 (14%)	
Dexamethasone ≥ 6 mg^‡^	136 (96%)	251 (97%)	0.60
*Other COVID-19 treatments*			
Tocilizumab	23 (16%)	45 (17%)	0.86
Convalescent plasma	6 (4%)	14 (5%)	0.77
Inhaled nitric oxide	12 (8%)	24 (9%)	0.92
Epoprostenol	29 (20%)	49 (19%)	0.83
Neuromuscular blockade	41 (29%)	83 (32%)	0.57
Baricitinib	2 (1%)	6 (2%)	0.72
*Investigational agents*			
Razuprotafib	–	10 (4%)	
Apremilast	–	31 (12%)	
Aviptadil	–	20 (8%)	
Celecoxib/Famotidine	–	18 (7%)	
Cenicriviroc	–	43 (17%)	
Cyclosporine	–	9 (3%)	
Cyproheptadine	–	6 (2%)	
IC14	–	40 (16%)	
Icatibant	–	38 (15%)	
Narsoplimab	–	22 (9%)	
Pulmozyme	–	21 (8%)	
28-day mortality	30 (21%)	71 (28%)	0.2
60-day mortality	36 (25%)	82 (32%)	0.22

Numbers are presented as n (%) unless otherwise stated

*BMI* body mass index, *LPM* liters per minute

*Determined via Welch’s t-test for normally distributed continuous variables, Wilcoxon rank-sum for non-normally distributed continuous variables, and Chi-squared test or Fisher’s exact test for categorical variables

^†^Using the WHO ordinal scale for COVID-19 severity; 5: hospitalized, noninvasive mechanical ventilation or high-flow nasal cannula (HFNC); 6: hospitalized, intubation and invasive mechanical ventilation (IMV); 7: hospitalized, IMV + additional support such as pressors or extracardiac membranous oxygenation

^‡^Completed course of steroid therapy prior to trial enrollment or receiving dexamethasone at the time of trial enrollment

### Baseline protein biomarkers and associations with outcomes

Baseline concentrations of several biomarkers were significantly associated with 28-day mortality. Specifically, increased concentrations of SARS-CoV-2 antigen, biomarkers of inflammation (IL-6, IL-8, IL-10, triggering receptor expressed on myeloid cells [TREM-1], sTNFR-1, interferon-gamma induced protein 10 [IP-10]), alveolar epithelial injury (soluble receptor for advanced glycation end products [sRAGE], surfactant protein D [SP-D]) and endothelial injury (thrombomodulin, intercellular adhesion molecule 1 [ICAM-1], and the ratio of Ang-2 to Ang-1 [Ang-2/Ang-1]) were significantly associated with increased odds of 28-day mortality (Additional file 2: Figure S2, Table S2).

After adjusting for confounders, SARS-CoV-2 antigen, IL-6, IL-8, IL-10, TREM-1, sTNFR-1, sRAGE, and thrombomodulin were still associated with 28-day mortality. In unadjusted analyses, all the biomarkers associated with 28-day mortality were also associated with increased odds of 60-day mortality (Additional file 2: Figure S2, Table S3). Additionally, increased concentrations of Ang-1, vascular endothelial growth factor (VEGF), and protein C were associated with a reduced odds of 60-day mortality. After adjusting for confounders, IL-6, IL-8, IL-10, IP-10, TREM-1, sTNFR-1, thrombomodulin, Ang-1, sRAGE, and SARS-CoV-2 antigen were still associated with 60-day mortality.

Given the impact of tocilizumab on inflammatory biomarkers, we performed a sensitivity analysis by removing the 68 patients (17%) who received tocilizumab during their hospitalization. We found similar associations of biomarkers with 28- and 60-day mortality, with some exceptions (Additional file 2: Figure S3). In the unadjusted analyses, ICAM-1 was no longer associated with 28-day mortality, and Ang-2 became significantly associated with 28- and 60-day mortality. In the adjusted analyses, IL-10 was no longer associated with 28-day mortality.

The same biomarkers associated with 60-day mortality were also associated with time to death and recovery (Additional file 2: Tables S4, S5). Additionally, protein C and Ang-1 were significantly associated with recovery. Results of extended Cox model analyses are provided in Additional file 2: Tables S6 and S7.

### Latent class analysis

We fit four models with increasing number of classes and found that a two-class model was the best fit for the data based on fit characteristics (Table 2). Using this model, 292 participants (73%) were categorized as Subtype 1 and 108 (27%) as Subtype 2. The average probability for most likely latent class membership was 0.96 for Subtype 1 and 0.93 for Subtype 2. Subtype 2 patients were older and more likely to be on invasive mechanical ventilation (IMV) at trial enrollment (33% vs 9%) (Table 3). Time from symptom onset, not included in the LCA, was similar between the two groups (Table 3). Subtype 2 patients were more likely to have comorbidities such as cerebrovascular disease, congestive heart failure, hypertension, diabetes, and chronic kidney disease (Additional file 2: Table S8).

**Table 2:** Fit statistics of latent class analysis results using clinical and protein biomarker data at study enrollment

Number of subtypes	BIC	Entropy	Number of participants per subtype				*P* value*
			*N*_1_	*N*_2_	*N*_3_	*N*_4_	
1	29,780		400				
2	29,454	0.83	292	108			0.01
3^†^	29,370	0.82	189	170	41		0.10
4^†^	29,328	0.83	152	132	75	41	0.11

*BIC* Bayesian Information Criterion

*Calculated using Vuong–Lo–Mendell–Rubin method to test whether a model with an additional class fits better than a model with one fewer class

^†^Model failed to replicate the maximum likelihood with up to 400 random starts

**Table 3:** Summary of patient characteristics by latent class analysis subtype assignment

	Subtype 1 (N = 292)	Subtype 2 (N = 108)	P-value*
**Variables included in LCA model**			
Age	57.7 ± 13.8	65.7 ± 13.5	< 0.0001
Male sex	192 (66%)	75 (69%)	0.56
BMI (median (IQR))	32 (28 to 37)	32 (27 to 38)	0.47
*Race*			0.91
Not White or Caucasian	88 (30%)	30 (28%)	
White or Caucasian	157 (54%)	57 (53%)	
Unknown	47 (16%)	21 (19%)	
*Respiratory support*			< 0.0001
≤ 15 LPM oxygen	32 (11%)	10 (9%)	
> 15 LPM oxygen	204 (70%)	51 (47%)	
Noninvasive mechanical ventilation	30 (10%)	10 (9%)	
Invasive Mechanical ventilation	26 (9%)	36 (33%)	
*Labs*^*†*^			
Platelets (10^9^/L)	275 ± 91	225 ± 84	< 0.0001
Bicarbonate (mmol/L)	25.1 ± 5.8	21.8 ± 3.3	< 0.0001
Hematocrit (%)	40 ± 5	36 ± 6	< 0.0001
Bilirubin (mg/dL)	0.5 (0.4 to 0.7)	0.6 (0.4 to 0.9)	0.13
Creatinine (mg/dL)	0.8 (0.7 to 1)	1.4 (1.1 to 2.3)	< 0.0001
WBC (10^9^/L)	8.7 (6.2 to 11.9)	10.9 (7.7 to 14.9)	0.0002
Neutrophil:Lymphocyte	8.9 (5.7 to 14.9)	14.9 (8.8 to 27.3)	< 0.0001
PTT (s)	28.7 (26 to 32)	33 (28.3 to 45.1)	< 0.0001
CRP (mg/L)	54.3 (13.7 to 129)	108 (21 to 169)	0.01
BNP (pg/mL)	71 (32 to 177)	563 (174 to 1477)	< 0.0001
*Biomarkers (median (IQR))*			
SARS-CoV-2 viral antigen protein (pg/mL)	732 (81 to 5,918)	1,358 (123 to 13,784)	0.01
Protein C (% normal)	118 (95 to 140)	89 (66 to 109)	< 0.0001
PAI-1 (ng/mL)	6.5 (4.4 to 9.3)	6.6 (4.7 to 13.3)	0.17
ICAM-1 (pg/mL)	660,243 (424,368 to 940,689)	724,537 (485,815 to 921,318)	0.44
sTNFR-1 (pg/mL)	2,492 (1,851 to 3,259)	4,757 (3,605 to 6,627)	< 0.0001
IL-6 (pg/mL)	15.5 (6.3 to 61.2)	28.9 (12.8 to 72.7)	< 0.0001
IL-8 (pg/mL)	11.4 (7.0 to 17.7)	14.8 (8.8 to 26.5)	0.0002
Ang-2 (pg/mL)	1,616 (1,035 to 2,552)	3,142 (1,948 to 5,272)	< 0.0001
sRAGE (pg/mL)	5,965 (3,272 to 10,643)	12,323 (7,368 to 18,280)	< 0.0001
IP-10 (pg/mL)	394 (175 to 646)	850 (364 to 1,870)	< 0.0001
VEGF (pg/mL)	42.7 (21.6 to 82.8)	28.2 (15.7 to 52.2)	0.0003
MMP-8 (pg/mL)	2,239 (1,302 to 4,046)	3,540 (1,990 to 6,337)	< 0.0001
SP-D (pg/mL)	15,883 (7,667 to 26,658)	20,851 (9,970 to 38,126)	0.005
Ang-1 (pg/mL)	8,208 (4,875 to 15,454)	5,745 (2,742 to 11,023)	0.0001
IL-18 (pg/mL)	401 (310 to 543)	544 (412 to 703)	< 0.0001
**Variables not included in LCA model**			
Time from symptom onset, days	8.8 ± 3.7	9.0 ± 5.3	0.70
Dexamethasone ≥ 6 mg^‡^	282 (97%)	105 (97%)	0.99
*Other COVID-19 treatments*			
Tocilizumab	56 (19%)	12 (11%)	0.08
Convalescent plasma	13 (5%)	7 (7%)	0.57
Inhaled nitric oxide	24 (8%)	12 (11%)	0.48
Epoprostenol	54 (19%)	24 (22%)	0.49
Neuromuscular blockade	82 (28%)	42 (39%)	0.05
Baricitinib	8 (3%)	0	0.11
*Investigational arm*			0.08
Apremilast	20 (7%)	11 (10%)	
Aviptadil	15 (5%)	5 (5%)	
Celecoxib/Famotidine	16 (5%)	2 (2%)	
Cenicriviroc	31 (11%)	12 (11%)	
Control	101 (35%)	41 (38%)	
Cyclosporine	9 (3%)	0	
Cyproheptadine	5 (2%)	1 (1%)	
IC14	30 (10%)	10 (9%)	
Icatibant	24 (8%)	14 (13%)	
Narsoplimab	19 (7%)	3 (3%)	
Pulmozyme	18 (6%)	3 (3%)	
Razuprotafib	4 (1%)	6 (6%)	
28-day mortality	57 (20%)	44 (41%)	< 0.0001
60-day mortality	69 (24%)	49 (45%)	< 0.0001

Numbers are presented as *n*(%) or mean ± SD unless stated otherwise

Ang = angiopoietin; BMI = body mass index; BNP = brain natriuretic peptide; CRP = C reactive protein; ICAM = intercellular adhesion molecule; IL = interleukin; IP = interferon-gamma induced protein; IQR = interquartile range. MMP = matrix metalloproteinase; PAI = plasminogen activator inhibitor; PTT = partial thromboplastin time; SP-D = surfactant protein D; sRAGE = soluble receptor for advanced glycation end products; sTNFR = soluble tumor necrosis factor receptor; TREM = triggering receptor expressed on myeloid cells; VEGF = vascular endothelial growth factor; WBC = white blood cell.

*Determined via Welch’s t-test for normally distributed continuous variables, Wilcoxon rank-sum for non-normally distributed continuous variables, and Chi-squared test or Fisher’s exact test for categorical variables

^†^Presented as mean ± SD or median(IQR) if variable demonstrated a skewed distribution

^‡^Completed course of steroid therapy prior to trial enrollment or receiving dexamethasone at the time of trial enrollment

**Fig. 1:**
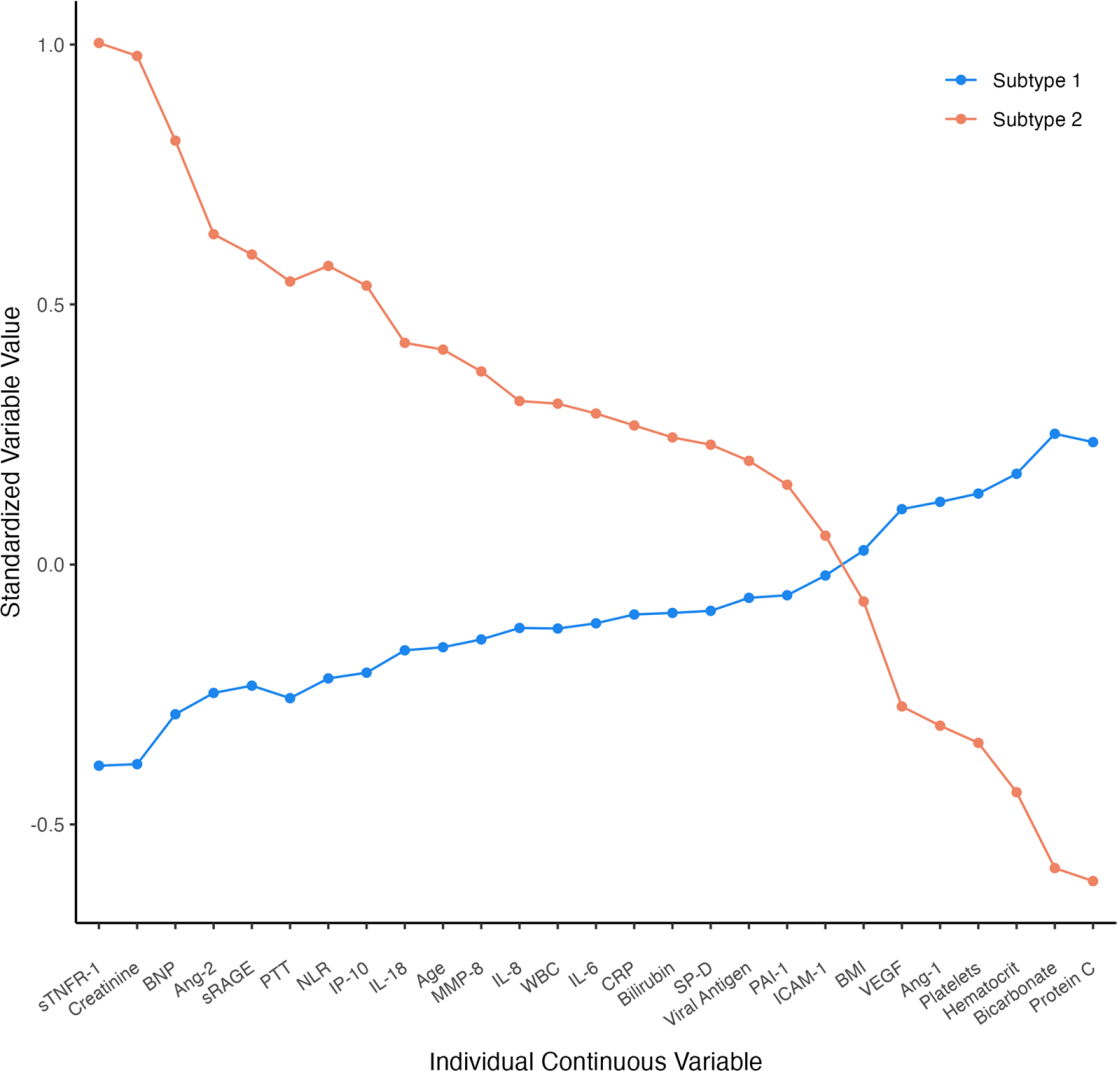
Differences in the standardized values of each continuous variable by subtype assignment. The variables are sorted based on the degree of separation between the two subtypes. A standardized value of + 1 signifies that the mean value for a given subtype was one standard deviation higher than the mean value in the cohort as a whole. Ang = angiopoietin; BMI = body mass index; BNP = brain natriuretic peptide; CRP = C reactive protein; ICAM1 = intercellular adhesion molecule-1; IL = interleukin; IP10 = interferon-gamma inducible protein of 10 kDa; MMP8 = matrix metalloproteinase-8; NLR = neutrophil to lymphocyte ratio; PAI1 = plasminogen activator inhibitor-1; PTT = partial thromboplastin time; RAGE = soluble receptor for advanced glycation end products; SPD = surfactant protein D; TNFR1 = tumor necrosis factor receptor-1; VEGF = vascular endothelial growth factors; WBC = white blood cell count

Compared to Subtype 1, Subtype 2 had higher baseline levels of sTNFR-1, creatinine, B-type natriuretic peptide (BNP), Ang-2, sRAGE, partial thromboplastin time (PTT), neutrophil to lymphocyte ratio, IP-10, IL-18, MMP-8, IL-8, white blood cell count (WBC), IL-6, C reactive protein (CRP), total bilirubin, SP-D, and SARS-CoV-2 antigen (Fig. 1**, **Table 3). ICAM-1, plasminogen activator inhibitor 1 (PAI-1), and BMI were not significantly different between the two groups. Subtype 2 had lower levels of protein C, bicarbonate, hematocrit, platelets, Ang-1, and VEGF. Creatinine, sTNFR-1, protein C, and bicarbonate were the most subtype separating variables. Given the strong association between SARS-CoV-2 antigen levels and outcomes in the literature, we conducted a sensitivity analysis by repeating LCA after excluding this analyte. There were no notable differences between the models though five patients switched subtypes (three became Subtype 1, and two became Subtype 2), suggesting that antigen levels were not a significant driver of subtype differences (Additional file 2: Figure S4).

**Fig. 2:**
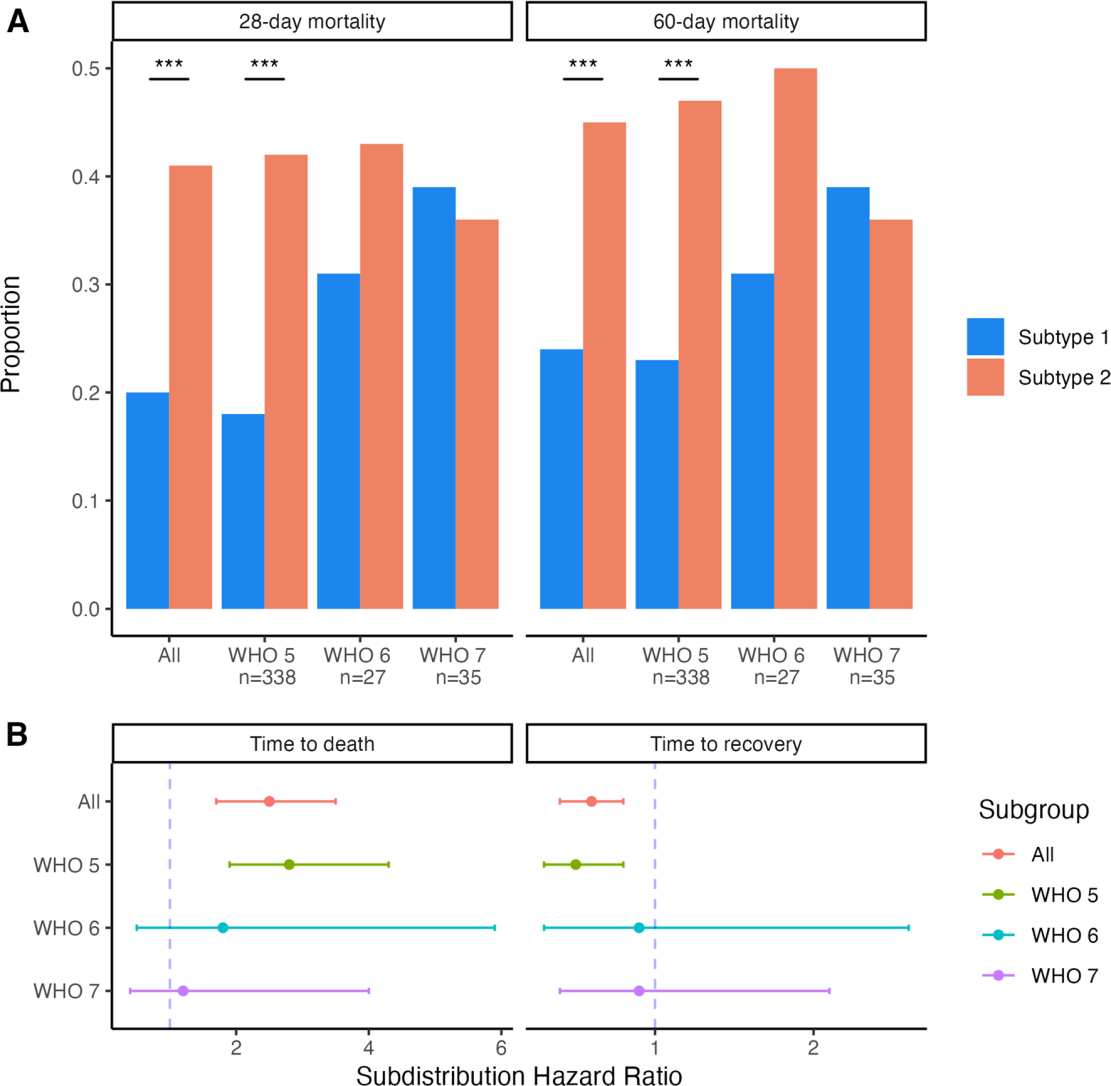
Clinical outcomes by latent class analysis (LCA) subtype assignment. **A** Mortality rates by subtype assignment stratified by WHO ordinal scale for COVID-19 severity upon trial enrollment; Within strata comparisons done using Chi-squared test or Fisher’s exact test where appropriate and p-value < 0.05 is depicted via Asterix. **B** Survival and recovery for Subtype 2 compared with Subtype 1 stratified by WHO ordinal scale for COVID-19 severity upon trial enrollment; For time to death, estimates are derived from Fine-Gray subdistribution hazard model with recovery as the competing event; For time to recovery, estimates are derived from Fine-Gray subdistribution hazard model with death as the competing event. ***P value < 0.001. WHO 5: hospitalized, noninvasive mechanical ventilation or high-flow nasal cannula (HFNC); WHO 6: hospitalized, intubation and invasive mechanical ventilation (IMV); WHO 7: hospitalized, IMV + additional support such as pressors or extracardiac membranous oxygenation

Subtype 2 designated participants had higher 28-day (41% vs 20%, p < 0.0001) and 60-day mortality (45% vs 24%, p < 0.0001) compared to Subtype 1 (Fig. 2**, **Table 3). Subtype 2 was associated with a subdistribution hazard ratio (SHR) of death of 2.5 (95%CI 1.7 to 3.5, p-value < 0.001) compared to Subtype 1 (Fig. 2, Additional file 2: Table S9). Similarly, Subtype 2 was associated with longer time to recovery (SHR 0.6, 95%CI 0.4 to 0.8, p-value < 0.001) (Fig. 2, Additional file 2: Table S9). When the study cohort was re-categorized to those not on IMV (WHO 5) and those on IMV or additional life support (WHO ≥ 6), the association of subtypes with all outcomes was only significant amongst those not on IMV (Fig. 2, Additional file 2: Table S9).

Additionally, we performed sensitivity analyses to ensure that associations between latent subtypes and clinical outcomes were not mediated by patients’ assigned treatment arms. We removed the 57 participants who received one of the four active study drugs for which outcome data from the I-SPY COVID Trial has not yet been published. The strong association of Subtype 2 designation with mortality at 28 days (42% vs 18%, p < 0.0001) and 60 days (46% vs 22%, p < 0.0001) persisted. Next, we adjusted Fine-Gray models for assigned treatment arm in the full cohort of 400 patients. Subtype 2 was still associated with a subdistribution hazard ratio (SHR) of death of 2.7 (95%CI 1.8 to 3.9, p-value < 0.001) and longer time to recovery (SHR 0.5, 95%CI 0.4 to 0.7, p-value < 0.001) compared to Subtype 1.

We used the parsimonious model described by Sinha et al [32] using IL-8, bicarbonate, and protein C levels to determine which patients in this cohort would have been classified as having hyper-inflammatory ARDS. Only one participant was classified as hyper-inflammatory using this approach, and this participant had a Subtype 2 designation.

### Longitudinal biomarker assessment

**Fig. 3:**
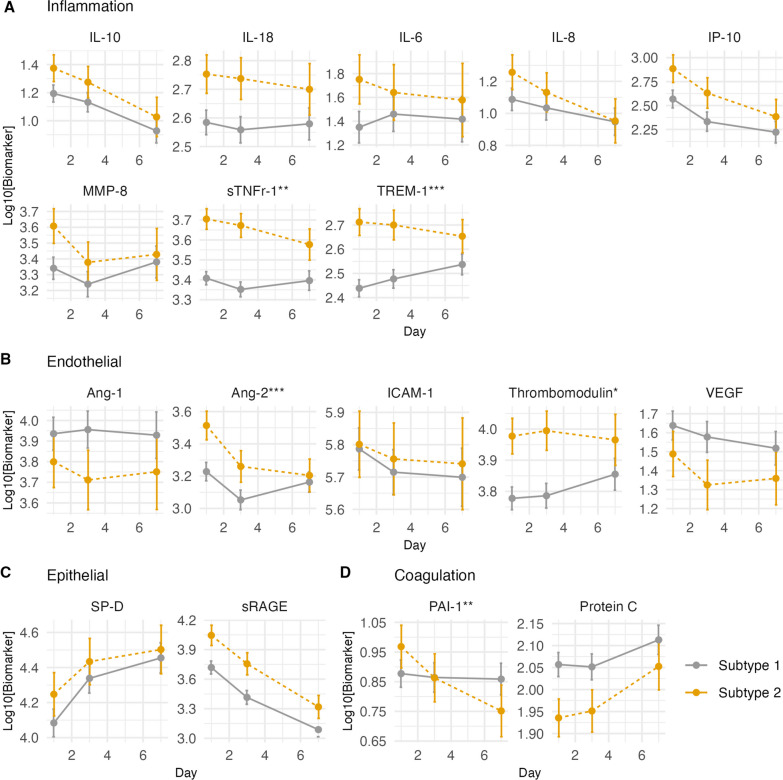
Change in log10 biomarker concentration over time by subtype assignment in the control arm (*N* = 142). **A** Biomarkers of inflammation, **B** endothelial injury, **C** alveolar epithelial injury, and **D** disordered coagulation. *Y*-axis is the log10 concentration of biomarker. Estimates with 95% confidence intervals derived from linear mixed effects models. Asterisks denote significant difference in the slope of biomarker change over time between Subtype 1 and Subtype 2 based on Chi-squared test of interaction. *p value < 0.05. **p value < 0.005. ***p value < 0.001

Longitudinal analyses were restricted to the 142 patients in the control arm. Pro-inflammatory biomarkers were higher across all time points in Subtype 2 compared to Subtype 1, and either remained elevated or slightly decreased over time (Fig. 3A). TREM-1 decreased over time in Subtype 2 but increased in Subtype 1, whereas sTNFR-1 decreased at a faster rate in Subtype 2 and remained relatively stable in Subtype 1. Of endothelial biomarkers, Ang-2 and thrombomodulin were higher and Ang-1 and VEGF were lower in Subtype 2 over time (Fig. 3B). Both epithelial biomarkers, SP-D and sRAGE, were higher across all time points in Subtype 2, with SP-D increasing and sRAGE decreasing in all patients (Fig. 3C). Of the coagulation biomarkers, PAI-1 and protein C, the latter was lower across all time points in Subtype 2 (Fig. 3D). PAI-1 decreased over time in Subtype 2 but remained stable in Subtype 1. A higher proportion of Subtype 2 designated patients died in the first week of enrollment compared with Subtype 1 (Additional file 2: Table S10).

## Discussion

In this secondary analysis of data from a randomized controlled trial, we found that participants with severe COVID-19 were characterized by significant biological heterogeneity. Using a combination of clinical data and protein biomarkers, we detected two latent subtypes of severe COVID-19 respiratory failure with significantly different compositions of biomarkers of inflammation, endothelial injury, alveolar epithelial injury, and disordered coagulation. Subtype 2 had significantly worse outcomes and was defined by higher baseline values of creatinine, sTNFR-1, BNP, Ang-2, and sRAGE and lower levels of protein C and bicarbonate among other differences. These subtypes differed from previously established COVID and non-COVID ARDS hyper- and hypo-inflammatory subtypes.

Though the subtypes, on average, demonstrate some differences in clinical parameters, no single clinical or biological parameter alone is sufficient for subtype designation. For instance, an older patient on IMV may appear clinically more worrisome but can still be designated as Subtype 1 based on the biological parameters incorporated into the model in our study. Indeed, a higher proportion of Subtype 2 designated patients were receiving IMV at trial enrollment. However, in stratified analyses, the subtypes’ prognostic value was most notable among those not on IMV. The lack of association with mortality among those on IMV or additional life support may be related to the small number of patients in these strata at study enrollment; nonetheless, the robust association with mortality among those not on IMV suggests that the subtypes capture pathobiology beyond the severity of acute respiratory failure. These findings have several implications. First, trials enrolling patients with severe COVID-19 as defined by respiratory failure status likely included a biologically heterogeneous patient population, which may explain inconsistent results in trials of corticosteroids and immunomodulators [6, 8, 12, 35–38] Our findings suggest that current clinical approaches to recruiting trial participants may be generating biologically heterogenous samples. Second, the treatment paradigm of patients with COVID-19, which primarily relies on the degree of respiratory failure, may be insufficient to identify those most likely to respond to therapies and at highest risk of poor outcomes. As steroids and immunomodulators have side effects, fine-tuning the target population most likely to respond to these medications would be desirable. Third, though most patients in our cohort did not meet the Berlin criteria for ARDS, our findings suggest that biological derangements leading to poor outcomes start earlier than the onset of IMV. In line with the new Global Definition of ARDS that includes those on high flow nasal oxygen [39], studying subtypes of non-COVID ARDS earlier in the course of disease and prior to the initiation of mechanical ventilation may both inform the natural trajectory of ARDS subtypes and offer an optimal window to deliver targeted therapies. Understanding early signs of biological pathways of injury is the first step toward the identification of treatable traits [40]. Future studies comparing our findings to those of patients with other types of severe viral pneumonia and/or ARDS from non-COVID related triggers will further aid in identifying shared pathways of injury that can be targeted for therapies.

The latent subtypes in this cohort of patients with hypoxemic respiratory failure had remarkable differences from the hyper- and hypo-inflammatory subtypes of COVID and non-COVID ARDS [20, 25]. Early in the pandemic, Sinha and colleagues discovered two latent subtypes of COVID-19 ARDS, as defined by the Berlin criteria, using LCA of readily available clinical data and IL-6. The authors then applied a validated clinical classifier model for non-COVID ARDS subtypes to phenotype their study cohort into hyper- and hypo-inflammatory ARDS. Over 80% of the participants in each of the two COVID ARDS subtypes overlapped with the corresponding non-COVID hypo- and hyper-inflammatory ARDS subtypes. In contrast, nearly all patients in our study cohort were categorized as hypo-inflammatory using the same parsimonious model. The lack of overlap of our subtypes with hyper- and hypo-inflammatory ARDS as compared with those of Sinha et al. is likely multi-factorial. First, most of our study cohort is patients with less severe respiratory failure than ARDS as defined by the Berlin Criteria. Our ability to still identify a latent subtype with over 40% mortality further supports the notion that biological signals of adverse outcomes are detectable earlier than the onset of IMV. Indeed, similar to hyper-inflammatory ARDS, Subtype 2 designated patients demonstrated higher levels of sTNFR-1 and lower levels of bicarbonate, protein C, and platelets compared to Subtype 1, suggesting similar pathways of injury. Biomarkers such as IL-6, IL-8, PAI-1, and ICAM-1, key to class designation in hyper- and hypo-inflammatory ARDS [20], were notably less class separating in severe COVID-19 subtypes, potentially rendering them less useful in phenotyping this patient population. Altogether, these differences may be related to differences in host–pathogen interactions over time in COVID and non-COVID respiratory failure as well as the impact of dexamethasone on biomarker concentrations in our study cohort. A recent study by Verhoef et al. performed latent profile analysis in a cohort of COVID-19 patients who presented to four different medical centers using residual blood samples collected during routine care [28]. The authors similarly found two latent profiles associated with significantly different clinical outcomes. The profiles demonstrated similar trends in the association between biomarkers of inflammation and endothelial injury as the subtypes in our study, although in contrast to our findings, IL-6 levels were the most important variable in profile designation. We are unable to directly compare the subtypes we observed to these latent profiles, given important differences between the cohorts (e.g., inclusion of outpatients in that cohort, variable corticosteroid usage) and the lack of a classifier model for the latent profiles; however, taken together, these two studies indicate that important biological heterogeneity is clearly present even in relatively milder and earlier forms of acute respiratory illness. More studies on patients presenting earlier in respiratory disease onset are warranted to validate these findings.

Patients with a Subtype 2 designation in our study were characterized by a higher degree of inflammation, alveolar epithelial injury, endothelial injury, and coagulation abnormalities. Interestingly, SARS-CoV-2 antigen level, though higher in Subtype 2, was less important to the model than the aforementioned biomarkers. The association between viral RNA in plasma with biomarkers of inflammation, injury, and poor outcomes in COVID-19 has been established [41–43], and studies have found a similar association between viral antigen levels and clinical outcomes [44, 45]. Though our analyses do not allow conclusive causal inference, one potential mechanism for the pattern of biomarker derangements in this cohort may be that a higher viral burden at the onset of disease leads to more severe initial lung injury, triggering both local and systemic inflammation that mediate poor outcomes. Further investigation of pathogenic differences in host response in those with acute hypoxemic respiratory failure are needed.

Not only were the two subtypes in this study biologically different at baseline, but this difference was maintained over the first 7 days of study enrollment. Subtype 2 was characterized by persistent inflammation, endothelial dysfunction, epithelial injury, and abnormal coagulation throughout the first week of trial enrollment. Some biomarker trajectories demonstrated a temporal trend toward convergence. Given similar rates of immunomodulator and dexamethasone use between the subtypes, the improving trend over time in Subtype 2 may have been in part due to an imbalance between those who recovered and those who died between the two subtypes during the first week of the study (Additional file 2: Table S9). Nonetheless, the pattern of persistent biomarker derangements in Subtype 2 raises important considerations. Baseline biomarker differences between the two subtypes persist beyond the immediate period in which the subtypes are defined, rendering them more likely to be clinically meaningful. For instance, high levels of TREM-1, a marker of innate immune response, have been associated with disease severity, duration of mechanical ventilation, and clinical outcomes in COVID-19 [46, 47]. Indeed, TREM-1 inhibition in those with severe COVID-19 demonstrated a trend toward reduction in 28-day mortality in a phase 2 randomized controlled trial [48]. Surprisingly, higher baseline TREM-1 levels did not predict beneficial response from therapy, suggesting that cross-sectional single biomarker measurements may not adequately capture treatment responsive subsets of patients. Similarly, a randomized, double-blind, placebo-controlled trial of oral imatinib, a tyrosine-kinase inhibitor, found that imatinib significantly reduced 90-day mortality in hospitalized adults with severe COVID-19 [49]. In secondary analyses, the effect of imatinib on mortality was fully mediated by a reduction in IL-6 levels, yet baseline plasma IL-6 concentration did not moderate the effect of imatinib on outcomes [50]. Indeed, using a panel of biomarkers, the authors found that a cluster of patients marked by increased levels of biomarkers of alveolar epithelial injury was the only one that experienced a meaningful mortality reduction with imatinib, suggesting that a complex interplay of alveolar epithelial injury, endothelial barrier integrity, and systemic inflammation is likely needed to experience mortality reduction from imatinib. Subtype 2 designated patients in our study appear to capture such a population of patients. The persistent dysregulation in these pathways and the differential mortality rates early in our study further suggest that interventions targeting these pathways of injury may have the highest value at the earliest timepoint of delivery. As we transition out of the pandemic, applying these insights to patients with non-COVID acute hypoxemic respiratory failure is worth consideration. Tools for real time subtyping in non-COVID ARDS are emerging [51] and capacity building studies are underway, laying the foundation for future trials to randomize patients based in part on biological subtypes. Understanding similarities and differences in biological heterogeneity between COVID and non-COVID respiratory failure is important to aid in targeting therapies to those with shared biological pathways of injury.

Our study has notable strengths. Patients in this cohort were recruited from a variety of academic and community hospitals across the United States, yielding a diverse cohort which enhances generalizability. Furthermore, participants were enrolled within a clinical trial with largely uniform clinical practices. The large panel of biomarkers in our study were carefully chosen a priori based on data from non-COVID ARDS as well as early findings in COVID-19. Our study also has limitations. Only participants with biospecimens available at day 1 were included, which may have introduced selection bias, although the clinical characteristics and outcomes of the patients without plasma were similar to those included. In the absence of a separate validation cohort, we did not attempt to develop a classifier model for pragmatic identification of these subtypes for research or clinical care. Lastly, given the high mortality rate in this cohort, informative missingness due to different survival rates between the subtypes limits any conclusions that can be drawn from longitudinal biomarker analyses.

## Conclusions

In this secondary analysis of data from a randomized controlled trial of hospitalized adults with severe COVID-19, we found evidence of two latent subtypes with divergent clinical outcomes. Subtype 2, comprising 27% of the cohort, was characterized by persistent inflammation, epithelial and endothelial injury, as well as disordered coagulation and had more than twice the mortality of Subtype 1. Latent biological heterogeneity may be contributing to negative findings in trials of pharmacotherapies. Early subtyping of patients with acute hypoxemic respiratory failure has the potential to identify biological pathways underlying clinical outcomes, reveal treatment-responsive subgroups of patients, and offer an earlier window for study of targeted therapies.

### Supplementary Information

**Additional file 1**. Supplemental methods.

**Additional file 2**. Supplemental tables and figures.

## Data Availability

The data reported in this manuscript is maintained by the study sponsor, Quantum Leap Healthcare Collaborative (QLHC). Source data supporting this manuscript is available to qualified investigators upon approval by QLHC and the ISPY COVID Data Access and Publications Committee. Requests can be initiated by contacting the corresponding author of this manuscript.

## Abbreviations

Ang: Angiopoietin
ARDS: Acute respiratory distress syndrome
BNP: B-type natriuretic peptide
COVID-19: Coronavirus disease 2019
CRP: C reactive protein
ELISA: Enzyme linked immunoassay methods
FDR: False discovery rate
ICAM-1: Intercellular adhesion molecule 1
IFN: Interferon
IL: Interleukin
IMV: Invasive mechanical ventilation
IP: Interferon-gamma induced protein
LCA: Latent class analysis
MMP: Matrix metalloproteinase
PAI-1: Plasminogen activator inhibitor 1
PEEP: Positive end expiratory pressure
PTT: Partial thromboplastin time
SARS-CoV-2: Severe acute respiratory syndrome coronavirus-2
SHR: Subdistribution hazard ratio
SP-D: Surfactant protein D
sRAGE: Soluble receptor for advanced glycation end products
sTNFR-1: Soluble tumor necrosis factor receptor 1
TNF: Tumor necrosis factor
TREM: Triggering receptor expressed on myeloid cells
VEGF: Vascular endothelial growth factor
WBC: White blood cell count
WHO: World Health Organization
