# Supplementary material for: Novel subtypes of severe COVID-19 respiratory failure based on biological heterogeneity: a secondary analysis of a randomized controlled trial
Source: Crit Care. 2024 Feb 21;28:56. doi: 10.1186/s13054-024-04819-0 (PMC10882728; doi:10.1186/s13054-024-04819-0)
Supplement: Supplementary file 1 — Additional file 1. Supplemental methods. [file 13054_2024_4819_MOESM1_ESM.docx]

*Study Design*

Data were obtained from the I-SPY COVID Trial, a multi-institutional phase 2 platform randomized controlled open-label trial to evaluate pharmacotherapies for severe COVID-19 (NCT04488081). The trial protocol and results for the first seven agents, none of which met predefined criteria for benefit, have been reported.^1,2^ In brief, newly hospitalized adults with SARS-CoV-2 requiring ≥6 liters/min supplemental oxygen were randomized to either a control arm receiving backbone therapy (Remdesivir and dexamethasone) or an investigational arm receiving backbone therapy plus a study drug. The trial’s primary endpoints were time to death and time to recovery, defined as ≥ 2 days at COVID level < 4 on the WHO COVID-19 ordinal scale for disease severity.^3^

*Assay Procedures*

Plasma was collected on days 1 (baseline, prior to administration of any active study drug), 3, and 7 following trial enrollment, stored at -80°C, then shipped and processed centrally at the University of California, San Francisco. Some protein biomarkers were measured on the following customized and previously validated multiplex Luminex panels: (1) IL-6, IL-8, Ang-2, RAGE; (2) ICAM-1, TNF RI; (3) SP-D, IL-10, IL-18, IP-10, TREM-1, MMP-8, Ang-1, VEGF, Thrombomodulin. Levels of protein C and PAI-1 are measured in duplicate using single analyte enzyme linked immunoassay methods (ELISA).

*Statistical Analysis*

Clinical data for these analyses were obtained from the I-SPY COVID Phase 2 Platform Randomized Controlled Trial of investigational agents for the treatment of severe COVID-19, released on May 13, 2022. The primary outcome for the present analysis was 28-day mortality. Secondary outcomes were 60-day mortality, time to death, and time to recovery. Biomarker values below the level of assay detection were replaced with half the lower limit of normal except for intracellular adhesion molecule 1 (ICAM-1), which was replaced with the lower limit of normal due to abnormal data distribution. Biomarkers were then log-transformed to approximate normal distribution. Biomarkers with minimum values <1 were shifted by 1 point prior to log transformation.

The association of each biomarker at baseline with mortality was tested using an unadjusted logistic regression model as well as a model adjusting for age, BMI, and level of respiratory support required at study enrollment. The covariates were selected based on evidence of significant association with primary outcomes of interest in the study cohort. P-values were adjusted for multiple comparisons using False Discovery Rate (FDR) cutoff of 0.05. Fine-Gray model for cumulative incidence function was used to estimate a subdistribution hazard ratio (SHR) for the association of each biomarker with time to death and time to recovery, as recovery and death were competing events in the clinical trial. The SHR provides the relative change in the rate of the occurrence of the outcome of interest in subjects who have not yet experienced that outcome (but who may have experienced the competing event). For example, SHR for recovery provides the relative change in the rate of recovery in subjects who have not yet recovered, but who may have experienced death. By maintaining deaths in the risk set of recovery, the model acknowledges that someone lost to death will never recover. The assumptions of subhazard proportionality were tested by introducing an interaction term between each biomarker of interest and time. For biomarkers with a non-linear proportional hazard over time, we fit an extended Cox model to estimate the cause-specific hazard of death over discrete time intervals since study enrollment.

A set of clinical and protein biomarker data were selected *a priori* to serve as class defining variables for LCA, a type of finite mixture modeling which assumes that “latent” classes exist within a multivariate distribution.^4^ We excluded two variables with more than 50% missingness (vasopressor use and troponin). Skewed data were log-transformed, and continuous variables were z-scaled. In accordance with standard LCA practices,^4^ three biomarkers (TREM1, Thrombomodulin, and IL-10) were removed due to high correlation with other existing biomarkers in the panel (sTNFR-1 and IP-10). Modeling was performed agnostic of clinical outcomes. The best fit model was selected based on the Bayesian Information Criteria (BIC), Entropy, and the Vuong-Lo-Mendell-Rubin (VLMR) test.^4^ An individual’s class assignment was determined by the highest probability of class membership (> 0.5).

We compared subtypes using Welch’s t-test, Wilcoxon rank-sum, or Pearson’s Chi-squared test depending on the distribution of the variable of interest. As noted previously, a Fine-Gray model for cumulative incidence function was used to estimate a SHR for the association of subtype with time to death and time to recovery. The association of each subtype with outcome was also described in subgroups stratified by initial disease severity (WHO COVID scale on admission). A validated parsimonious model using interleukin (IL)-8, bicarbonate, and protein C was used for comparison with established non-COVID-19 related hyper-inflammatory and hypo-inflammatory ARDS LCA subtypes.^5^

Using biomarker measurements from days 1, 3, and 7 since trial enrollment, we fit linear mixed effects models to determine whether subtype assignment was associated with differences in biomarker trajectory over time. Fixed effects included biomarker plasma value and the interaction of day of plasma sample collection with baseline subtype assignment. Each patient was modeled as a random factor with patient-specific collection day coefficients. Longitudinal analyses were restricted to the control arm due to the unmeasurable confounding effect of various agents used in the investigational arm of the study.

All data analysis was conducted in R version 4.2.2 and STATA version 17.0. LCA modeling was done in MPlus version 8.8.

1. Consortium ISC. Report of the first seven agents in the I-SPY COVID trial: a phase 2, open label, adaptive platform randomised controlled trial. EClinicalMedicine 2023;58:101889. DOI: 10.1016/j.eclinm.2023.101889.

2. Files DC, Matthay MA, Calfee CS, et al. I-SPY COVID adaptive platform trial for COVID-19 acute respiratory failure: rationale, design and operations. BMJ Open 2022;12(6):e060664. DOI: 10.1136/bmjopen-2021-060664.

3. Organization WH. COVID-19 Therapeutic Trial Synopsis. (<https://www.who.int/publications/i/item/covid-19-therapeutic-trial-synopsis>).

4. Sinha P, Calfee CS, Delucchi KL. Practitioner's Guide to Latent Class Analysis: Methodological Considerations and Common Pitfalls. Crit Care Med 2021;49(1):e63-e79. DOI: 10.1097/CCM.0000000000004710.

5. Sinha P, Delucchi KL, McAuley DF, O'Kane CM, Matthay MA, Calfee CS. Development and validation of parsimonious algorithms to classify acute respiratory distress syndrome phenotypes: a secondary analysis of randomised controlled trials. The Lancet Respiratory medicine 2020;8(3):247-257. DOI: 10.1016/S2213-2600(19)30369-8.
