# Supplementary material for: Novel subtypes of severe COVID-19 respiratory failure based on biological heterogeneity: a secondary analysis of a randomized controlled trial
Source: Crit Care. 2024 Feb 21;28:56. doi: 10.1186/s13054-024-04819-0 (PMC10882728; doi:10.1186/s13054-024-04819-0)
Supplement: Supplementary file 2 — Additional file 2. Supplemental tables and figures. [file 13054_2024_4819_MOESM2_ESM.docx]

Table S1. Baseline characteristics of trial participants included and excluded from the analyses.

|  | **Excluded (N=468)** | | **Included**  **(N=400)** | | **P-value*** |
| --- | --- | --- | --- | --- | --- |
| **Age (years)** | 58.8 + 14.7 | | 59.8 + 14.2 | | 0.29 |
| **Sex (Female)** | 171 (36%) | | 133 (33%) | | 0.35 |
| **BMI** | 31.8 (27.2 to 37.7) | | 31.8 (27.7 to 37.2) | | 0.77 |
| **COVID-19 severity -admission**^†^ |  |  | | 0.40 | |
| WHO 5 | 410 (88%) | 338 (84%) | |  | |
| WHO 6 | 27 (6%) | 27 (7%) | |  | |
| WHO 7 | 31 (7%) | 35 (9%) | |  | |
| **28-day mortality** | 95 (20%) | | 101 (25%) | | 0.10 |
| **60-day mortality** | 122 (26%) | | 118 (29%) | | 0.29 |

Presented as mean + SD or median (IQR) if variable demonstrated a skewed distribution.

* Determined via Welch’s t-test for normally distributed continuous variables, Wilcoxon rank-sum for non-normally distributed continuous variables, and Chi-squared test or Fisher’s exact test for categorical variables.

† WHO 5: hospitalized, noninvasive mechanical ventilation or high-flow nasal cannula (HFNC); WHO 6: hospitalized, intubation and invasive mechanical ventilation (IMV); WHO 7: hospitalized, IMV + additional support such as pressors or extracardiac membranous oxygenation.

Table S2. Association of baseline protein biomarker concentration with 28-day mortality.

|  | **28-day mortality** | | | | |
| --- | --- | --- | --- | --- | --- |
|  | univariate | | multivariate^†^ | | |
|  | OR (95%CI) | P-value* | | OR (95%CI) | P-value* |
| **Inflammation** |  |  | |  |  |
| IL-6 | 1.8 (1.3 to 2.6) | 0.003 | | 1.8 (1.2 to 2.7) | 0.009 |
| IL-8 | 4.2 (2.0 to 8.9) | <0.001 | | 4.0 (1.7 to 9.4) | 0.009 |
| IL-10 | 3.1 (1.6 to 6.0) | 0.003 | | 2.8 (1.3 to 5.9) | 0.02 |
| IL-18 | 1.9 (0.7 to 5.4) | 0.23 | | 2.5 (0.8 to 8.0) | 0.19 |
| IP-10 | 2.0 (1.3 to 3.2) | 0.006 | | 1.6 (0.9 to 2.7) | 0.14 |
| MMP-8 | 1.0 (0.5 to 1.7) | 0.87 | | 0.8 (0.4 to 1.5) | 0.53 |
| sTNFR-1 | 23 (7.4 to 70) | <0.001 | | 8.8 (2.4 to 32) | 0.009 |
| TREM-1 | 28 (8.6 to 93) | <0.001 | | 7.9 (2.0 to 31) | 0.009 |
| **Epithelial Injury** |  |  | |  |  |
| sRAGE | 5.5 (2.6 to 11.3) | <0.001 | | 4.3 (2.0 to 9.5) | 0.005 |
| SP-D | 2.5 (1.4 to 4.5) | 0.006 | | 1.6 (0.8 to 3.1) | 0.24 |
| **Endothelial injury** |  |  | |  |  |
| Ang-1 | 0.4 (0.3 to 0.7) | 0.003 | | 0.5 (0.3 to 0.8) | 0.02 |
| Ang-2 | 1.9 (0.9 to 3.9) | 0.09 | | 1.6 (0.7 to 3.8) | 0.31 |
| Ang-2/Ang-1 | 4.5 (1.6 to 13.1) | 0.009 | | 3.2 (0.9 to 11.8) | 0.14 |
| ICAM-1 | 2.7 (1.1 to 6.4) | 0.04 | | 3.2 (1.1 to 8.9) | 0.05 |
| VEGF | 0.6 (0.3 to 1.0) | 0.07 | | 0.6 (0.3 to 1.2) | 0.20 |
| Thrombomodulin | 28 (7.4 to 104) | <0.001 | | 8.4 (2.0 to 34) | 0.009 |
| **Disordered Coagulation** |  |  | |  |  |
| Protein C | 0.3 (0.1 to 1.3) | 0.12 | | 1.5 (0.3 to 7.6) | 0.63 |
| PAI-1 | 0.7 (0.3 to 1.9) | 0.52 | | 0.5 (0.2 to 1.6) | 0.30 |
| **SARS-CoV-2 Viral Antigen** | 1.4 (1.1 to 1.8) | 0.003 | | 1.4 (1.1 to 1.8) | 0.009 |

Ang = angiopoietin; ICAM = intercellular adhesion molecule; IL = interleukin; IP = interferon-gamma induced protein; MMP = matrix metalloproteinase; PAI = plasminogen activator inhibitor; SP-D = surfactant protein D; sRAGE = soluble receptor for advanced glycation end products; sTNFR = soluble tumor necrosis factor receptor; TREM = triggering receptor expressed on myeloid cells; VEGF = vascular endothelial growth factor.

Estimates based on logistic regression.

* P-values adjusted for multiple comparisons using False Discovery Rate (FDR).

† Model adjusted for age, BMI, and level of respiratory support delivered on admission.

Table S3. Association of baseline protein biomarker concentration with 60-day mortality.

|  | **60-day mortality** | | | | |
| --- | --- | --- | --- | --- | --- |
|  | univariate | | multivariate^†^ | |  |
|  | OR (95%CI) | P-value* | OR (95%CI) | P-value* |  |
| **Inflammation** |  |  |  |  |  |
| IL-6 | 1.8 (1.3 to 2.4) | 0.002 | 1.8 (1.2 to 2.6) | 0.02 |  |
| IL-8 | 3.8 (1.9 to 7.9) | <0.001 | 3.6 (1.6 to 8.2) | 0.02 |  |
| IL-10 | 3.3 (1.7 to 6.3) | <0.001 | 3.0 (1.5 to 6.4) | 0.02 |  |
| IL-18 | 1.6 (0.6 to 4.1) | 0.38 | 2.0 (0.7 to 6.1) | 0.25 |  |
| IP-10 | 2.2 (1.4 to 3.5) | 0.001 | 1.9 (1.1 to 3.1) | 0.04 |  |
| MMP-8 | 1.0 (0.6 to 1.7) | 0.92 | 0.8 (0.4 to 1.6) | 0.58 |  |
| sTNFR-1 | 13 (4.4 to 36) | <0.001 | 5.0 (1.5 to 17) | 0.02 |  |
| TREM-1 | 20 (6.5 to 62) | <0.001 | 6.0 (1.6 to 22) | 0.02 |  |
| **Epithelial Injury** |  |  |  |  |  |
| sRAGE | 5.8 (2.9 to 11.7) | <0.001 | 4.5 (2.1 to 9.6) | 0.002 |  |
| SP-D | 2.1 (1.2 to 3.7) | 0.01 | 1.4 (0.7 to 2.6) | 0.34 |  |
| **Endothelial injury** |  |  |  |  |  |
| Ang-1 | 0.5 (0.3 to 0.8) | 0.006 | 0.5 (0.3 to 0.9) | 0.04 |  |
| Ang-2 | 1.7 (0.9 to 3.3) | 0.16 | 1.6 (0.7 to 3.7) | 0.31 |  |
| Ang-2/Ang-1 | 4.2 (1.5 to 12) | 0.01 | 3.3 (0.9 to 12.2) | 0.10 |  |
| ICAM-1 | 2.4 (1.0 to 5.4) | 0.05 | 2.7 (1.0 to 7.0) | 0.06 |  |
| VEGF | 0.5 (0.3 to 0.9) | 0.02 | 0.5 (0.3 to 0.9) | 0.06 |  |
| Thrombomodulin | 18 (5.3 to 63) | <0.001 | 6.4 (1.7 to 24) | 0.02 |  |
| **Disordered Coagulation** |  |  |  |  |  |
| Protein C | 0.1 (0.03 to 0.6) | 0.009 | 0.5 (0.1 to 2.2) | 0.34 |  |
| PAI-1 | 0.5 (0.2 to 1.4) | 0.23 | 0.4 (0.1 to 1.2) | 0.14 |  |
| **SARS-CoV-2 Viral Antigen** | 1.4 (1.1 to 1.7) | 0.003 | 1.4 (1.1 to 1.7) | 0.02 |  |

Ang = angiopoietin; ICAM = intercellular adhesion molecule; IL = interleukin; IP = interferon-gamma induced protein; MMP = matrix metalloproteinase; PAI = plasminogen activator inhibitor; SP-D = surfactant protein D; sRAGE = soluble receptor for advanced glycation end products; sTNFR = soluble tumor necrosis factor receptor; TREM = triggering receptor expressed on myeloid cells; VEGF = vascular endothelial growth factor.

Estimates based on logistic regression.

* P-values adjusted for multiple comparisons using False Discovery Rate (FDR).

† Model adjusted for age, BMI, and level of respiratory support delivered on admission.

OR = odds ratio.

Table S4. Association of baseline protein biomarker concentration with time to death.

|  | **Time to death** | | | | |
| --- | --- | --- | --- | --- | --- |
|  | univariate | | multivariate^†^ | |  |
|  | SHR* (95%CI) | P-value | SHR* (95%CI) | P-value |  |
| **Inflammation** |  |  |  |  |  |
| IL-6 | 1.6 (1.3 to 2) | <0.001 | 1.6 (1.2 to 2.1) | 0.002 |  |
| IL-8 | 3 (1.7 to 5.2) | <0.001 | 2.8 (1.5 to 5.2) | 0.001 |  |
| IL-10 | 2.8 (1.7 to 4.6) | <0.001 | 2.1 (1.2 to 3.7) | 0.01 |  |
| IL-18 | 1.7 (0.8 to 3.5) ^‡^ | 0.17 | 2.2 (1.0 to 5.0) ^‡^ | 0.06 |  |
| IP-10 | 2.1 (1.5 to 2.9) | <0.001 | 1.6 (1.1 to 2.4) | 0.02 |  |
| MMP-8 | 0.9 (0.5 to 1.4) | 0.50 | 0.8 (0.5 to 1.3) | 0.42 |  |
| sTNFR-1 | 9.8 (4.2 to 23) ^‡^ | <0.001 | 4.2 (1.6 to 11) ^‡^ | 0.003 |  |
| TREM-1 | 13 (5.1 to 33) ^‡^ | <0.001 | 4.7 (1.6 to 13) ^‡^ | 0.004 |  |
| **Epithelial Injury** |  |  |  |  |  |
| sRAGE | 5.3 (2.8 to 9.9) | <0.001 | 3.4 (1.8 to 6.2) | <0.001 |  |
| SP-D | 1.7 (1.0 to 2.7) | 0.04 | 1.2 (0.7 to 2.0) | 0.58 |  |
| **Endothelial injury** |  |  |  |  |  |
| Ang-1 | 0.6 (0.4 to 0.8) | 0.004 | 0.7 (0.4 to 1) | 0.04 |  |
| Ang-2 | 1.5 (0.8 to 2.7) | 0.17 | 1.3 (0.7 to 2.6) | 0.38 |  |
| Ang-2/Ang-1 | 2.8 (1.3 to 6.3) | 0.01 | 2.0 (0.8 to 4.7) | 0.12 |  |
| ICAM-1 | 2.1 (1.1 to 4) | 0.02 | 2.3 (1.1 to 4.6) | 0.02 |  |
| VEGF | 0.6 (0.4 to 0.9) | 0.01 | 0.7 (0.4 to 1.0) | 0.06 |  |
| Thrombomodulin | 12 (3.7 to 40) ^‡^ | <0.001 | 3.9 (1.2 to 13) ^‡^ | 0.02 |  |
| **Disordered Coagulation** |  |  |  |  |  |
| Protein C | 0.2 (0.1 to 0.5) | 0.002 | 0.6 (0.2 to 1.9) | 0.41 |  |
| PAI-1 | 0.7 (0.3 to 1.6) | 0.42 | 0.6 (0.3 to 1.4) ^‡^ | 0.25 |  |
| **SARS-CoV-2 Viral Antigen** | 1.5 (1.2 to 1.7) | <0.001 | 1.4 (1.1 to 1.6) | 0.001 |  |

Ang = angiopoietin; ICAM = intercellular adhesion molecule; IL = interleukin; IP = interferon-gamma induced protein; MMP = matrix metalloproteinase; PAI = plasminogen activator inhibitor; SP-D = surfactant protein D; sRAGE = soluble receptor for advanced glycation end products; sTNFR = soluble tumor necrosis factor receptor; TREM = triggering receptor expressed on myeloid cells; VEGF = vascular endothelial growth factor.

SHR = subdistribution hazard ratio

* Estimates from Fine-Gray subdistribution hazard model with recovery as the competing event.

† Model adjusted for age, BMI, and level of respiratory support delivered on admission.

‡ Model violates proportional subdistribution hazards assumption. The effect of biomarker on outcome varies over time so summary estimate reported is not accurate.

Table S5. Association of baseline protein biomarker concentration with time to recovery.

|  | **Time to recovery** | | | | |
| --- | --- | --- | --- | --- | --- |
|  | univariate | | multivariate^†^ | |  |
|  | SHR* (95%CI) | P-value | SHR* (95%CI) | P-value |  |
| **Inflammation** |  |  |  |  |  |
| IL-6 | 0.6 (0.5 to 0.8) ^‡^ | <0.001 | 0.6 (0.5 to 0.8) | <0.001 |  |
| IL-8 | 0.5 (0.3 to 0.7) | 0.001 | 0.6 (0.4 to 0.9) | 0.02 |  |
| IL-10 | 0.3 (0.2 to 0.5) ^‡^ | <0.001 | 0.4 (0.2 to 0.5) ^‡^ | <0.001 |  |
| IL-18 | 0.7 (0.4 to 1.1) | 0.12 | 0.7 (0.4 to 1.2) | 0.21 |  |
| IP-10 | 0.5 (0.3 to 0.6) | <0.001 | 0.5 (0.4 to 0.7) | <0.001 |  |
| MMP-8 | 1.0 (0.7 to 1.4) ^‡^ | 0.98 | 1.2 (0.8 to 1.6) ^‡^ | 0.41 |  |
| sTNFR-1 | 0.2 (0.1 to 0.4) | <0.001 | 0.3 (0.2 to 0.6) | 0.001 |  |
| TREM-1 | 0.2 (0.1 to 0.4) | <0.001 | 0.4 (0.2 to 0.8) | 0.007 |  |
| **Epithelial Injury** |  |  |  |  |  |
| sRAGE | 0.3 (0.2 to 0.4) ^‡^ | <0.001 | 0.3 (0.2 to 0.5) ^‡^ | <0.001 |  |
| SP-D | 0.8 (0.6 to 1.0) | 0.09 | 1.0 (0.7 to 1.3) | 0.80 |  |
| **Endothelial injury** |  |  |  |  |  |
| Ang-1 | 1.6 (1.1 to 2.1) | 0.004 | 1.4 (1.1 to 1.9) | 0.02 |  |
| Ang-2 | 0.9 (0.6 to 1.3) | 0.49 | 1.1 (0.7 to 1.6) | 0.80 |  |
| Ang-2/Ang-1 | 0.5 (0.3 to 1.1) | 0.10 | 0.7 (0.3 to 1.5) | 0.39 |  |
| ICAM-1 | 0.6 (0.4 to 0.8) | 0.005 | 0.6 (0.4 to 0.8) | 0.003 |  |
| VEGF | 1.4 (1.0 to 1.8) | 0.04 | 1.3 (1.0 to 1.7) | 0.06 |  |
| Thrombomodulin | 0.4 (0.2 to 0.6) ^‡^ | <0.001 | 0.5 (0.3 to 0.9) ^‡^ | 0.02 |  |
| **Disordered Coagulation** |  |  |  |  |  |
| Protein C | 4.2 (1.8 to 9.7) ^‡^ | 0.001 | 2.9 (1.2 to 7) ^‡^ | 0.02 |  |
| PAI-1 | 0.9 (0.5 to 1.4) | 0.53 | 1.0 (0.6 to 1.7) | 0.99 |  |
| **SARS-CoV-2 Viral Antigen** | 0.7 (0.6 to 0.8) | <0.001 | 0.7 (0.6 to 0.8) | <0.001 |  |

Ang = angiopoietin; ICAM = intercellular adhesion molecule; IL = interleukin; IP = interferon-gamma induced protein; MMP = matrix metalloproteinase; PAI = plasminogen activator inhibitor; SP-D = surfactant protein D; sRAGE = soluble receptor for advanced glycation end products; sTNFR = soluble tumor necrosis factor receptor; TREM = triggering receptor expressed on myeloid cells; VEGF = vascular endothelial growth factor.

SHR = subdistribution hazard ratio.

* Estimates from Fine-Gray subdistribution hazard model with recovery as the competing event.

† Model adjusted for age, BMI, and level of respiratory support delivered on admission.

‡ Model violates proportional subdistribution hazards assumption. The effect of biomarker on outcome varies over time so summary estimate reported is not accurate.

Table S6. Association of baseline protein biomarker with time to death for time-varying biomarkers.

|  | **Time to death**^†^ | | | | | | |
| --- | --- | --- | --- | --- | --- | --- | --- |
|  | 2 weeks | | 4 weeks | | 6 weeks | |  |
|  | HR* (95%CI) | P-value | HR* (95%CI) | P-value | HR* (95%CI) | P-value |  |
| TREM-1 | 10.1 (1.9 to 54) | 0.007 | 2.2 (0.5 to 9.3) | 0.29 | 0.5 (0 to 13) | 0.69 |  |
| sTNFR-1 | 11.3 (2.4 to 53) | 0.002 | 2.3 (0.6 to 8.7) | 0.21 | 0.3 (0.02 to 5) | 0.40 |  |
| Thrombomodulin | 13.8 (2.4 to 78) | 0.003 | 1 (0.3 to 4.5) | 0.81 | 0.5 (0 to 5.2) | 0.55 |  |
| IL-18 | 5.9 (1.4 to 26) | 0.02 | 1.7 (0.5 to 5.9) | 0.40 | 0.45(0 to 8.2) | 0.59 |  |

IL = interleukin; sTNFR = soluble tumor necrosis factor receptor; TREM = triggering receptor expressed on myeloid cells.

HR = hazard ratio

* Estimates from extended Cox regression model.

† Model adjusted for age, BMI, and level of respiratory support delivered on admission.

Table S7. Association of baseline protein biomarker with time to recovery for time-varying biomarkers.

|  | **Time to recovery**^†^ | | | | | | | | | |
| --- | --- | --- | --- | --- | --- | --- | --- | --- | --- | --- |
|  |  | Period 1 ^‡^ | | Period 2 | | | Period 3 | | |  |
|  | Interval | HR* (95%CI) | P-value | HR* (95%CI) | P-value | HR* (95%CI) | | P-value |  |  |
| Protein C | 2 weeks | 3.5 (1.2 to 10) | 0.02 | 6.7 (0.9 to 52) | 0.07 | 0.5 (0 to 14) | | 0.66 |  |  |
| sRAGE | 7 days | 0.2 (0.1 to 0.3) | <0.001 | 0.5 (0.3 to 0.9) | 0.03 | 0.7 (0.3 to 1.6) | | 0.43 |  |  |
| IL-10 | 7 days | 0.2 (0.1 to 0.3) | <0.001 | 0.6 (0.3 to 1.1) | 0.08 | 0.7 (0.3 to 1.7) | | 0.46 |  |  |
| Thrombomodulin | 7 days | 0.4 (0.2 to 0.9) | 0.02 | 0.6 (0.2 to 1.5) | 0.26 | 1.4 (0.4 to 5.8) | | 0.61 |  |  |
| MMP-8 | 7 days | 1.0 (0.6 to 1.6) | 0.86 | 1.1 (0.6 to 2.0) | 0.65 | 1.4 (0.7 to 3.1) | | 0.34 |  |  |

IL = interleukin; MMP = matrix metalloproteinase; sRAGE = soluble receptor for advanced glycation end products.

HR = hazard ratio

* Estimates from extended Cox regression model.

† Model adjusted for age, BMI, and level of respiratory support delivered on admission.

‡ Optimal interval for each biomarker was selected based on plotting the Schoenfeld residuals over time from a cox proportional hazards model for each biomarker.

Table S8. Comorbidities by latent class subtype assignment.

|  | **Subtype 1  (N=292)** | **Subtype 2 (N=108)** | **P-value*** |
| --- | --- | --- | --- |
| **Comorbidities** |  |  |  |
| Cerebrovascular disease | 9 (3%) | 12 (11%) | 0.004 |
| Congestive heart failure | 11 (4%) | 14 (13%) | 0.002 |
| Diabetes | 93 (32%) | 50 (46%) | 0.01 |
| Chronic kidney disease | 17 (6%) | 30 (28%) | 0.01 |
| End stage kidney disease | 2 (0.7%) | 0 | 1 |
| Dialysis | 1 (0.3%) | 1 (0.9%) | 0.5 |
| Hypertension | 152 (52%) | 76 (70%) | 0.002 |
| Liver disease – mild | 4 (1%) | 1 (0.9%) | 1 |
| Liver disease – moderate to severe | 2 (0.7%) | 0 | 1 |
| Myocardial infarction | 5 (2%) | 6 (6%) | 0.08 |
| Peripheral vascular disease | 4 (1%) | 4 (4%) | 0.05 |
| Chronic Lung disease | 55 (19%) | 24 (22%) | 0.5 |
| Chronic rheumatologic disease | 15 (5%) | 6 (6%) | 1 |

Numbers are presented as n(%) or mean + SD unless stated otherwise.

* Determined via Chi-squared test or Fisher’s exact test for categorical variables.

Table S9. Clinical outcomes by latent class analysis subtype assignment.

|  | **Subtype 1  (N=292)** | **Subtype 2 (N=108)** | **P-value*** |
| --- | --- | --- | --- |
| **28-day mortality**, n(%) |  |  |  |
| All patients | 57 (20%) | 44 (41%) | <0.001 |
| WHO 5^§^ (n = 338) | 48 (18%) | 30 (42%) | <0.001 |
| WHO 6 (n = 27) | 4 (31%) | 6 (43%) | 0.80 |
| WHO 7 (n = 35) | 5 (39%) | 8 (36%) | 1 |
| WHO > 6 (n = 62) | 9 (35%) | 14 (39%) | 0.94 |
| **60-day mortality**, n(%) |  |  |  |
| All patients | 69 (24%) | 49 (45%) | <0.001 |
| WHO 5^§^ (n = 338) | 60 (23%) | 34 (47%) | <0.001 |
| WHO 6 (n = 27) | 4 (31%) | 7 (50%) | 0.53 |
| WHO 7 (n = 35) | 5 (39%) | 8 (36%) | 1 |
| WHO > 6 (n = 62) | 9 (35%) | 15 (42%) | 0.85 |
| **Time to death**^†^, SHR (95% CI) |  |  |  |
| All patients | Ref | 2.5 (1.7 to 3.5) | <0.001 |
| WHO 5 (n = 338) | Ref | 2.8 (1.9 to 4.3) | <0.001 |
| WHO 6 (n = 27) | Ref | 1.8 (0.5 to 5.9) | 0.33 |
| WHO 7 (n = 35) | Ref | 1.2 (0.4 to 4.0) | 0.73 |
| WHO > 6 (n = 62) | Ref | 1.4 (0.6 to 3.4) | 0.36 |
| **Time to recovery**^‡^, SHR (95% CI) |  |  |  |
| All patients | Ref | 0.6 (0.4 to 0.8) | <0.001 |
| WHO 5 (n = 338) | Ref | 0.5 (0.3 to 0.8) | 0.001 |
| WHO 6 (n = 27) | Ref | 0.9 (0.3 to 2.6) | 0.90 |
| WHO 7 (n = 35) | Ref | 0.9 (0.4 to 2.1) | 0.83 |
| WHO > 6 (n = 62) | Ref | 0.9 (0.5 to 1.8) | 0.83 |

SHR = subdistribution hazard ratio; Ref = reference.

* P-values derived from Chi-squared test for categorical outcomes and the Wald Chi-squared test for estimates from Fine-Gray subdistribution hazard model.

† Estimate derived from Fine-Gray subdistribution hazard model with recovery as the competing event.

‡ Estimate derived from Fine-Gray subdistribution hazard model with death as the competing event.

§ Using the WHO ordinal scale for COVID-19 severity; 5: hospitalized, noninvasive mechanical ventilation or high-flow nasal cannula (HFNC); 6: hospitalized, intubation and invasive mechanical ventilation (IMV); 7: hospitalized, IMV + additional support such as pressors or extracardiac membranous oxygenation.

Table S10. Mortality in the first 7 days compared to later by subtype.

|  | **Subtype 1**  **(N = 292)** | **Subtype 2**  **(N = 108)** | **P-value*** |
| --- | --- | --- | --- |
| **Outcomes < day 7**, n (%) |  |  |  |
| Died | 5 (2%) | 8 (7%) | 0.001 |
| Recovered | 78 (27%) | 15 (14%) | 0.01 |
| Censored | 9 (3%) | 2 (2%) | 1 |
| **Outcomes > day 7**, n(%) |  |  |  |
| Died | 61 (21%) | 42 (39%) | 0.002 |
| Recovered | 109 (37%) | 35 (32%) | 0.08 |
| Censored | 30 (10%) | 6 (1%) | 0.11 |

* P-value calculated using the Chi-squared test or Fisher’s exact test.


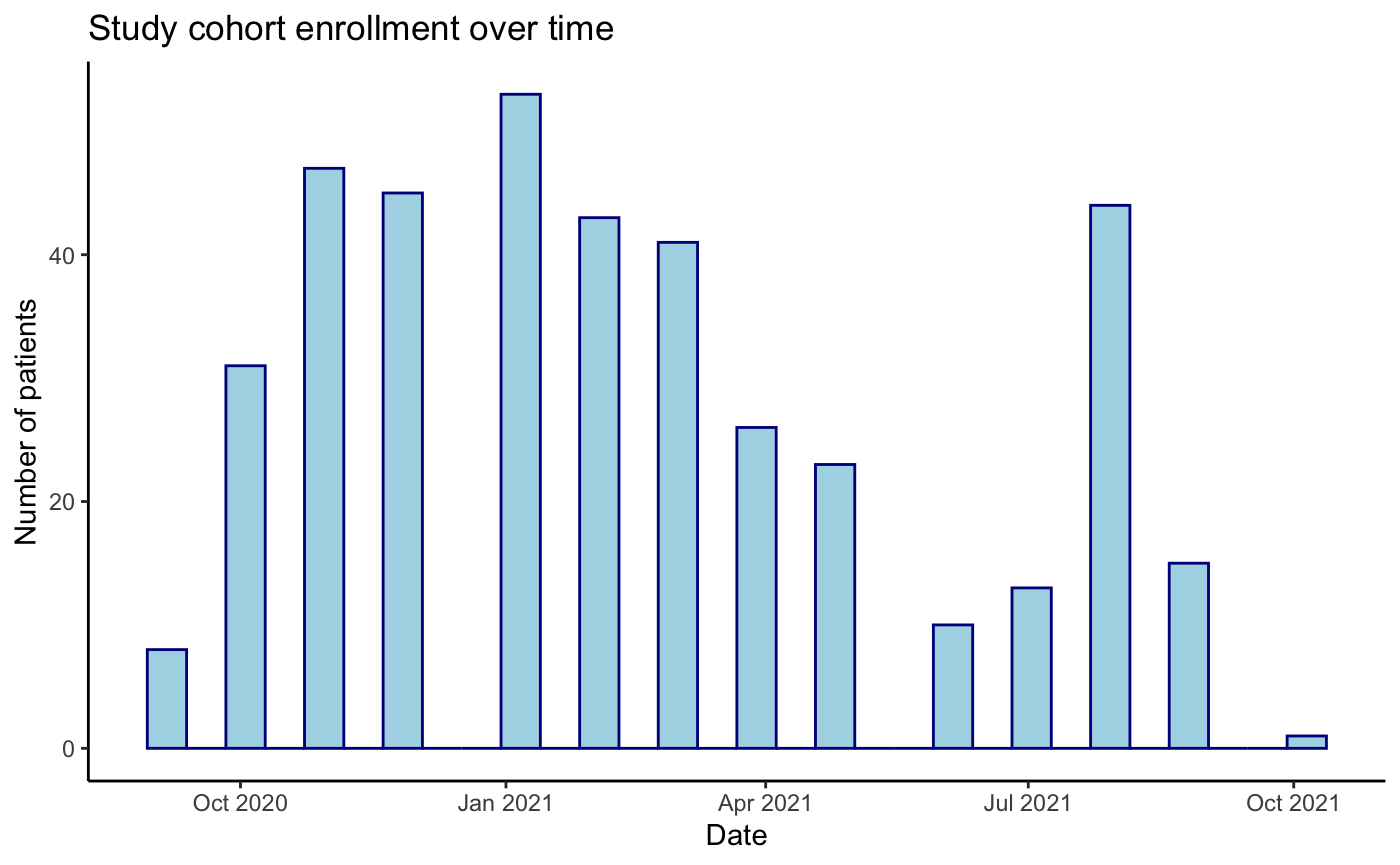


**Figure S1.** Distribution of enrollment date over time of the 400 study participants.


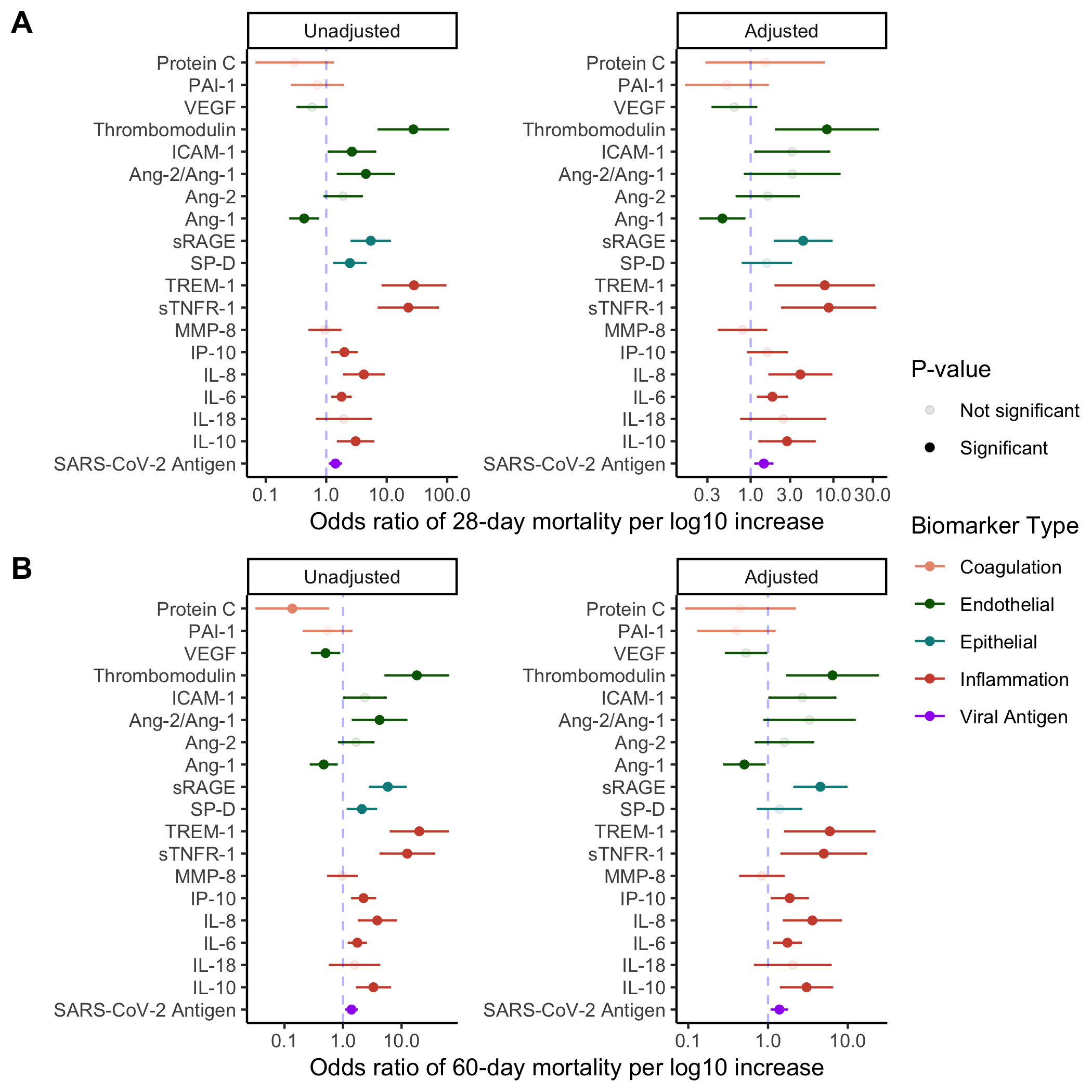


**Figure S2**. Association of baseline protein biomarker concentration with 28-day (panel A) and 60-day (panel B) mortality. Odds ratio and 95% confidence interval estimates based on logistic regression. Covariates in the adjusted analyses are age, BMI, and degree of respiratory support required at study enrollment. P-values adjusted for multiple comparisons using false discovery rate (FDR). Ang = angiopoietin; ICAM = intercellular adhesion molecule; IL = interleukin; IP = interferon-gamma induced protein; MMP = matrix metalloproteinase; PAI = plasminogen activator inhibitor; SP-D = surfactant protein D; sRAGE = soluble receptor for advanced glycation end products; sTNFR = soluble tumor necrosis factor receptor; TREM = triggering receptor expressed on myeloid cells; VEGF = vascular endothelial growth factor.


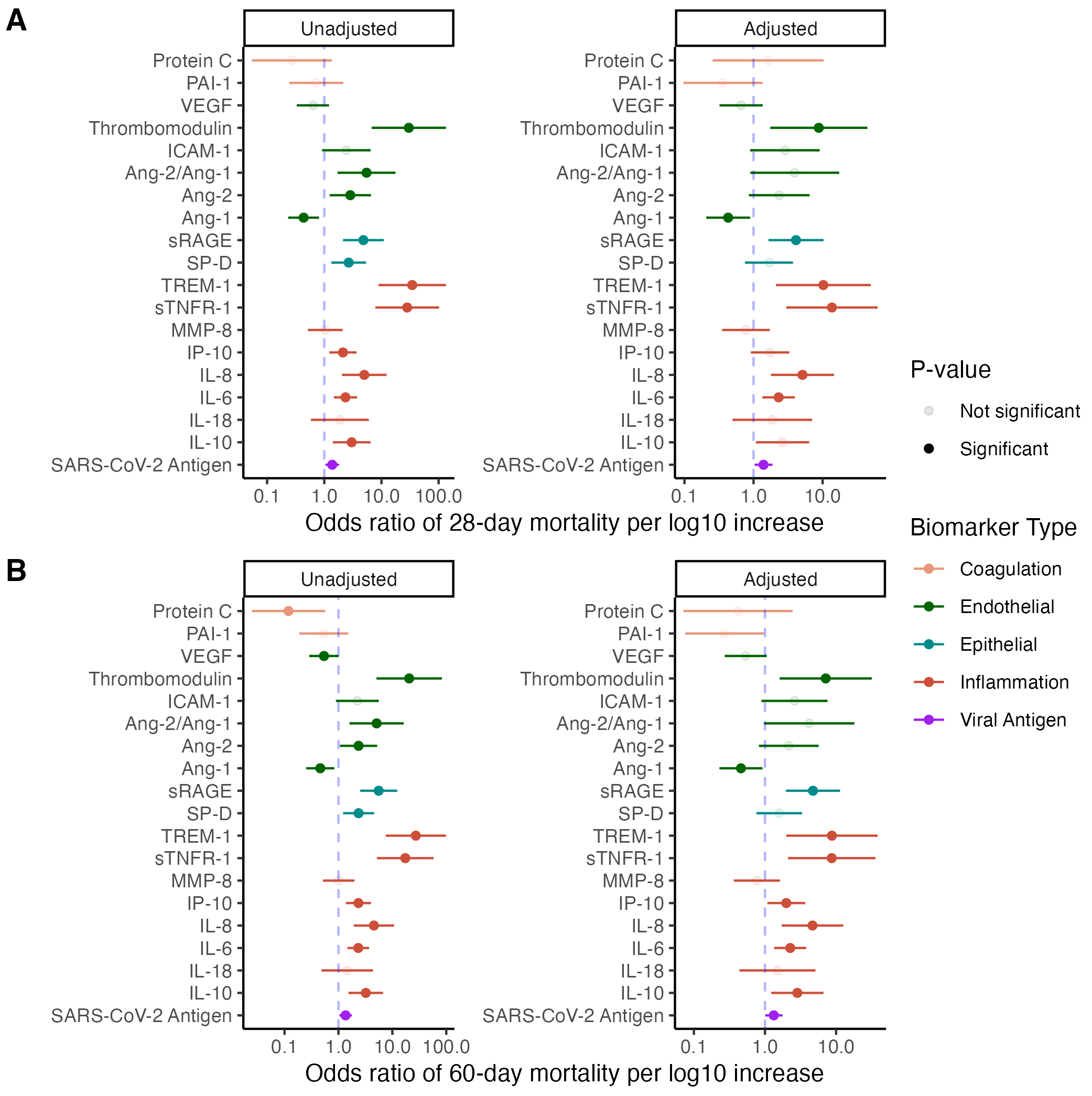


**Figure S3**. Association of baseline protein biomarker concentration with 28-day (panel A) and 60-day (panel B) mortality among patients who did not receive tocilizumab (N = 332). Odds ratio and 95% confidence interval estimates based on logistic regression. Covariates in the adjusted analyses are age, BMI, and degree of respiratory support required at study enrollment. P-values adjusted for multiple comparisons using false discovery rate (FDR). Ang = angiopoietin; ICAM = intercellular adhesion molecule; IL = interleukin; IP = interferon-gamma induced protein; MMP = matrix metalloproteinase; PAI = plasminogen activator inhibitor; SP-D = surfactant protein D; sRAGE = soluble receptor for advanced glycation end products; sTNFR = soluble tumor necrosis factor receptor; TREM = triggering receptor expressed on myeloid cells; VEGF = vascular endothelial growth factor.


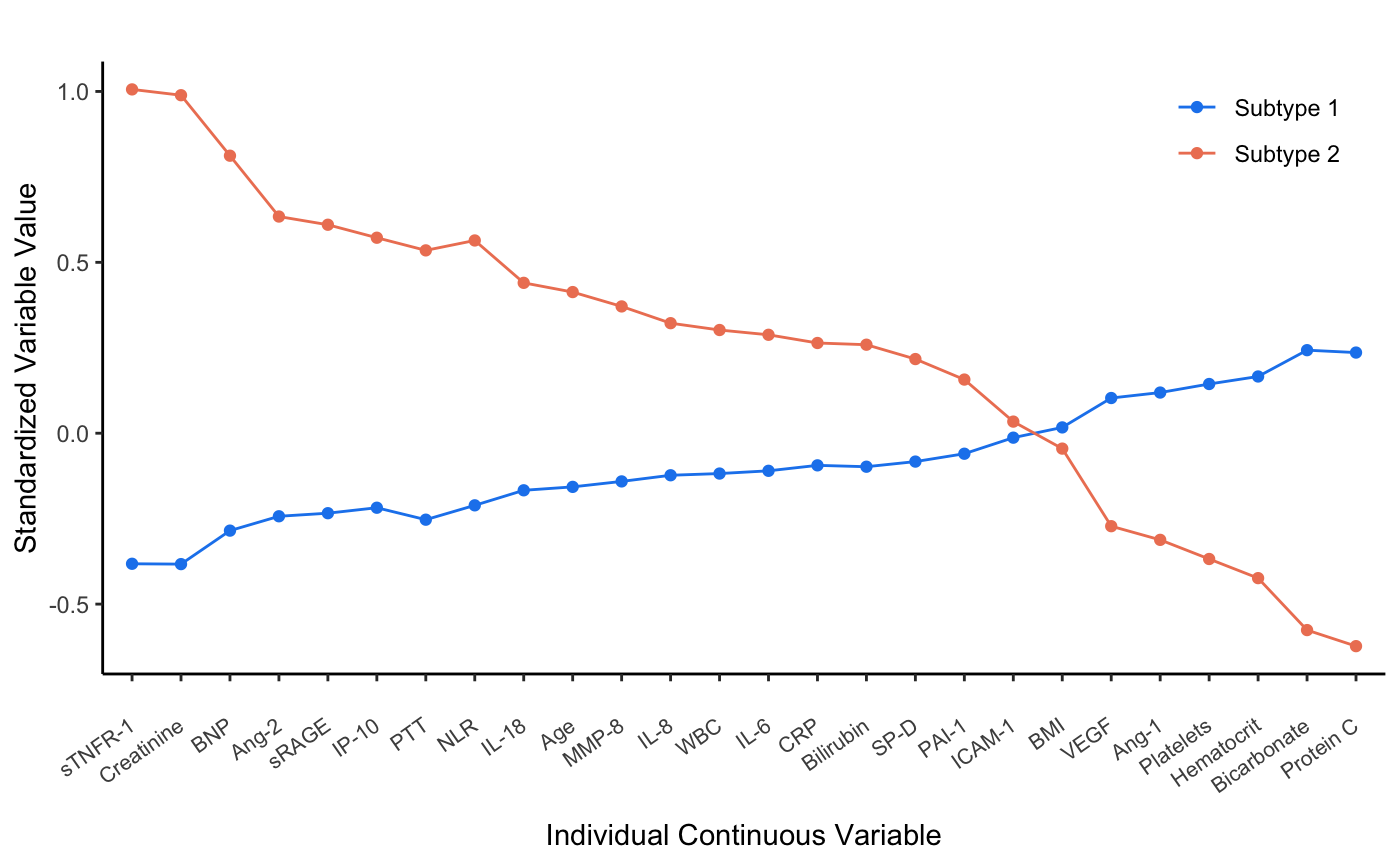


**Figure S4**. Differences in the standardized values of each continuous variable by subtype assignment after excluding viral antigen levels as a variable in the latent class analysis model. The variables are sorted based on the degree of separation between the two subtypes. A standardized value of +1 signifies that the mean value for a given subtype was one standard deviation higher than the mean value in the cohort as a whole.

Ang = angiopoietin; BMI = body mass index; BNP = brain natriuretic peptide; CRP = C reactive protein; ICAM1 = intercellular adhesion molecule-1; IL = interleukin; IP10 = interferon-gamma inducible protein of 10kDa; MMP8 = matrix metalloproteinase-8; PAI1 = plasminogen activator inhibitor-1; PTT = partial thromboplastin time; RAGE = soluble receptor for advanced glycation end products; SPD = surfactant protein D; TNFR1 = tumor necrosis factor receptor-1; VEGF = vascular endothelial growth factors; WBC = white blood cell count.
